# Epidemiology and disease characteristics of myelofibrosis: a comparative analysis between Italy and global perspectives

**DOI:** 10.3389/fonc.2024.1382872

**Published:** 2024-07-24

**Authors:** Massimo Breccia, Francesca Palandri, Nicola Polverelli, Morena Caira, Michela Berluti, Giuseppe A. Palumbo, Valerio De Stefano

**Affiliations:** ^1^ Dipartimento di Medicina Traslazionale e di Precisione, Sapienza Università, Roma, Italy; ^2^ IRCCS Azienda Ospedaliero-Universitaria di Bologna, Istituto di Ematologia Seragnoli, Bologna, Italy; ^3^ Unit of Blood Diseases and stem cell transplantation, ASST Spedali Civili di Brescia, University of Brescia, Brescia, Italy; ^4^ Medical Department, AbbVie Srl, Roma, Italy; ^5^ Dipartimento di Scienze Mediche Chirurgiche e Tecnologie Avanzate “G. F. Ingrassia”, Università di Catania, Catania, Italy; ^6^ Sezione di Ematologia, Dipartimento di Scienze Radiologiche ed Ematologiche, Università Cattolica, Fondazione Policlinico Universitario Agostino Gemelli IRCCS, Roma, Italy

**Keywords:** CALR, JAK2, MPL, myelofibrosis, myeloproliferative neoplasms, secondary fibrosis

## Abstract

Myelofibrosis (MF) is a clonal disorder of hematopoietic stem cells characterized by altered bone marrow function and fibrosis. The aim of this narrative review is to report on the most recent epidemiologic data and to discuss features of MF and current strategies for the management of this condition in clinical practice. MF features covered by our review will include: characteristics of patients with MF; myeloproliferative and myelodepletive phenotypes; MF-associated thrombosis and bleeding; risk of infections; prefibrotic and overt PMF; secondary MF. Finally, we will discuss a few aspects of MF management in clinical practice and suggest strategies for its optimization and standardization. The focus of our paper is on Italy, but relevant data from other countries will also be reviewed.

## Introduction

Myelofibrosis (MF) is a clonal disorder of hematopoietic stem cells characterized by altered bone marrow function and fibrosis (1, 2). According to the World Health Organization (WHO) classification updated in 2016 and refined in 2022 using the International Consensus Classification (ICC), primary myelofibrosis (PMF) belongs to the group of myeloproliferative neoplasms (MPNs) (2–7). MF can develop also in patients with essential thrombocythemia (ET) and polycythemia vera (PV) (secondary MF, or post-ET myelofibrosis and post-PV myelofibrosis, respectively). Patients with MF have a reduced life expectancy compared with the general population and experience a substantial symptom burden due mainly to constitutional symptoms and splenomegaly; common complications of MF include infections, thrombosis, bleeding, and leukemic transformation (2, 8–10).

The discovery in 2005 of the Janus kinase 2 (JAK2) V617F somatic mutation, a gain-of-function mutation that constitutively activates the JAK2 kinase leading to hyperresponsiveness of myeloid cells to cytokines, was a breakthrough in the field of MPNs (11–13). In subsequent years, two additional driver mutations in the genes *MPL* (thrombopoietin receptor) and *CALR* (calreticulin), as well as other mutations, were identified (2). These discoveries have greatly advanced the diagnosis of MPNs, and somatic mutations of *JAK2*, *MPL* and *CALR* are important criteria for the diagnosis of PMF, ET and PV according to the WHO/ICC classifications (3–6). Targeted therapies that inhibit JAK2 have also become available for MF, including ruxolitinib (approved by the European Medicines Agency (EMA) in 2013) and fedratinib (approved by the EMA in 2021) (2, 5).

MF is a rare condition. According to a meta-analysis of epidemiologic studies of MPNs published before 2014, and comprising mostly populations from Europe and North America, annual incidence rates of PMF ranged from 0.22 to 0.99 per 100,000 (14). As the diagnosis and classification of MPNs have significantly evolved in recent years, there is a need for updated epidemiologic data.

The aim of this narrative review is to summarize our knowledge of the epidemiology and main features of MF and to describe current strategies for the management of this condition. To this purpose, we will first present the main data about MF incidence, prevalence, and survival; we will then briefly review recent literature about the main characteristics of the MF population and about distinct disease phenotypes and entities. Finally, we will discuss issues of MF management in clinical practice. The focus of our paper is on Italy, but relevant data from other countries will be also reviewed.

## Methods

PubMed was searched with combinations of the following terms: “myeoloproliferative neoplasm”, “myelofibrosis”, “secondary myelofibrosis”, “polycythemia vera”, “thrombocythemia” AND “epidemiology”, “prevalence”, “incidence”, “survival rates”; other terms used for refining the search were “observational study”, “population-based study”. The time frame considered was 2016-2022 (i.e., after the 2016-update of the WHO classification), but the publication year alone did not limit the inclusion of a study in the present review. As Italy was the focus of the initial search, the term “AND Italy” was also included in a subset of searches. Given the paucity of epidemiological data on myelofibrosis in Italy, we have included other populations. In the period considered, we were able to identify relevant literature on the European, North American, African, Asian and Australian populations, while we were unable to find significant data for the South American population. Retrieved articles were first screened based on their titles; the abstracts of articles identified as potentially relevant were checked and the full text version of those articles that were confirmed as relevant was obtained. Criteria of relevance included: size of the MPN/MF population, the source of the data, study design, and the availability of data specific for MF. Abstracts from international congresses were also included. Further literature was retrieved from the reference lists of the articles identified by the search of PubMed. In addition, information on registries and ongoing programs/initiatives addressing MPNs/MF was found via internet searches. The term “MF” is used throughout this paper as an umbrella term that includes PMF, post-PV MF, and post-ET MF if the accuracy is not reported.

## Epidemiology of myelofibrosis

### Incidence, prevalence, survival rates

An overview of studies that have investigated the epidemiology of MF is provided in **Table 1**. Studies are listed according to the country considered, starting from Europe (15–20), followed by North America (21–25), Australia/New Zealand (26, 27), and Asia (28–30); within each area, studies are listed in chronological order based on the publication year. Most studies analyzed data about MPNs cases, with MF accounting usually for < 20% of the cases. The time frames considered by the analyses were highly variable; the most recent upper limit of the time frames was 2017, meaning that in most studies MPNs were not diagnosed according to the 2016-updated WHO classification (3, 7). Only the literature review by Moulard et al. and the analysis of the US Survey, Epidemiology, and End Results (SEER) database by Verstovsek et al. were exclusively devoted to MF/PMF (16, 23). Overall, the findings of these studies suggest that age-standardized incidence rates of PMF range from 0.3 to 0.8 per 100,000 person-years (19–21, 23, 25, 26, 29), the incidence is higher in men than women (21, 22, 26), and survival rates at 5 years from diagnosis are included between 50% and 58% (17, 26, 30). Among MPNs, PMF is associated with the worst survival rates (21); survival is better in women than in men (21, 30), and in people aged < 60 years than in people aged ≥ 60 years (21). The relatively high survival reported by Soyer et al. might have been due to the fact that > 70% of the PMF population analyzed belonged to the low or intermediate-1 risk categories (18).

**Table 1:** Summary of epidemiologic data about myelofibrosis.

Study	Country	Period	Population	Incidence/ Prevalence	Survival/ Mortality	Disease Classification
Bari et al., 2007 (15)	Italy	up to 2006	▪ Modena Cancer Registry ▪ Pts with chronic myeloproliferative disorders ▪ n=380; 41% ET, 30% PV, 20% MF	▪ ASR of chronic myeloproliferative disorders, 3.2/100,000 ▪ Crude incidence rate, 6.6/100,000	▪ 5-yr survival, 88% ▪ Better survival for ET and PV vs other subtypes	WHO 2001
Moulard et al., 2014 (16)	European Union	2000-2012	▪ Comprehensive literature review ▪ MPN data from 11 articles, 3 registries, 2 databases (Orphanet, RARECARE)	▪ Annual incidence rate of MF, 0.1-1.0/100,000 ▪ Prevalence of MF, 2.3/100,000 (Orphanet); 0.51/100,000/15 yrs (RARECARE)	na	na
Roaldsnes et al., 2016 (17)	Norway	1993-2012	▪ Cancer Registry of Norway ▪ Pts with MPN ▪ n=2453, 17.4% MF	▪ Age-adjusted annual incidence MF, 0.2-0.5/100,000 ▪ Prevalence MF, 3.0/100,000 (as of December 2011	▪ 5-yr survival MF, 58.1%	na
Soyer et al., 2017 (18)	Turkey	1987-2014	▪ MPN pts referring to 9 centers ▪ n=708, 13.5% with PMF	na	▪ 10-yr survival PMF, 82.5%	WHO
Hultcrantz et al., 2020 (19)	Sweden	2000-2014	▪ Swedish Cancer Register and Swedish Blood Cancer Register ▪ Pts with MPN ▪ n=6281, 740 (11.8%) with PMF	▪ Age-standardized incidence PMF, 0.52/100,000 person-yrs	na	PSVG and WHO
Solans et al., 2022 (20)	Spain	2002-2013	▪ 13 cancer registries ▪ n=17,522 ▪ Pts with MPN, 457 (2.6%) with PMF	▪ Crude incidence rate PMF, 0.36/100,000 person-yrs ▪ ASR PMF, 0.41/100,000	na	na
Srour et al., 2016 (21)	USA	2001-2012	▪ SEER database ▪ n=31,904 ▪ Pts with MPN, 2988 (9.4%) with PMF	▪ Incidence rate PMF, 3.1/10^6^ person-yrs ▪ In males, 4.1/10^6^ person-yrs ▪ In females, 2.3/10^6^ person-yrs	▪ Worst 5-yr relative survival of PMF among classical Ph- MPNs ▪ 5-yr relative survival better for pts aged < 60 yrs than pts aged ≥ 60 yrs ▪ 5-yr relative survival better for females than males	WHO 2008
Shallis et al., 2021 (22)	USA	2001-2016	▪ SEER database	▪ Age-adjusted incidence of PMF/100,000 person-yrs: 0.02 for age < 40 yrs; 0.1 for 40-49 yrs; 0.4 for 50-59 yrs; 1.1 for 60-69 yrs; 1.9 for 70-79 yrs; 2.0 for > 80 yrs ▪ Incidence generally higher in men than women	na	na
Verstovsek et al., 2022 (23)	USA	2002-2016	▪ SEER database ▪ n=4214 ▪ Pts with PMF	▪ Incidence rate PMF, 0.44/100,000 person-yrs	▪ 5-yr mortality PMF, 51%	na
Szuber et al., 2019 (24)	USA	1967-2017	▪ All consecutive pts with MPNs at Mayo Clinic ▪ n=3023, 1282 with PMF (42.4%)	na	▪ Overall survival for PMF pts, 4.4 yrs ▪ Significantly worse leukemia-free survival for PMF versus ET and PV	WHO 2016
Heppner et al., 2019 (25)	Canada	2011-2015	▪ Retrospective analysis of the database of a major Canadian cancer cytogenetics lab ▪ Patients with MPN	▪ Crude incidence PMF, 0.56/100,000 person-yrs ▪ Age-standardized incidence PMF, 0.80/100,000 person-yrs	na	WHO 2008
Baade et al., 2019 (26)	Australia	2003-2014	▪ Australian Cancer Database ▪ n=8604 ▪ Pts with MPN, 1799 (20.9%) with PMF	▪ Age-standardized incidence rate PMF, 4.5/10^6^ population ▪ 6.0/10^6^ population in males ▪ 3.2/10^6^ population in females	▪ 5-yr survival PMF, 50.1%	na
Varghese et al., 2021 (27)	New Zealand	2010-2017	▪ New Zealand Cancer Registry ▪ n=787 ▪ Pts with MPN, 153 (19.3%) with PMF^a^	▪ Annual (2017) incidence rate PMF, 0.92/100,000	▪ PMF mortality rate (over 2 yr-follow-up), 44.7%	na
Byun et al., 2017 (28)	Korea	2004-2013	▪ Korean National Health Insurance and Health Insurance Review and Assessment Service databases ▪ Patients with MPNs	▪ PMF prevalence (2004–2013), 0.5-0.9/100,000 ▪ PMF annual incidence rate, 0.3-0.5/100,000	na	na
Htun et al., 2022 (29)	Singapore	1968-2017	▪ Singapore Cancer Registry ▪ n=2557 ▪ Pts with MPN, 134 (5.2%) with PMF	▪ Age standardized incidence rate (2013-2017) PMF, 0.43/100,000	▪ 5-yr relative survival ratio compared with general population, 0.53 for PMF	na
Yap et al., 2022 (30)	Malaysia	2009-2015	▪ National MPN Registry ▪ n=774, ▪ Pts with MPN, 8.9% with PMF	na	▪ Mortality rate PMF, 60.9% (as of December 2018) ▪ 5-yr overall survival PMF, 53% (females, 60%; males, 46.7%)	PVSG criteria and WHO 2016

ASR, age standardized rate; ET, essential thrombocythemia; KCD, Korean classification disease; MF, myelofibrosis; MPN, myeloproliferative neoplasm; na, not available; PMF, primary myelofibrosis; pts, patients; PV, polycythemia vera; PVSG, polycythaemia vera study group yr, year.

a PMF patients entered in the registry since 2014.

On the whole, this overview highlights the paucity of epidemiological data for MF and the lack in many countries of standardized data reporting, as already pointed out by Moulard et al. in their comprehensive review of the epidemiologic literature published in 2014 (16). Epidemiologic data about MF in Italy are lacking. The Italian estimates reported in **Table 1** were made based on the 2001-WHO classification (15). To the best of our knowledge, updated Italian data, based on the latest WHO classifications are not available; also, there were no Italian data among the European studies reviewed by Moulard and coworkers (16).

The study based on the Cancer Registry of Norway revealed a gradual increase in the age-adjusted annual incidence rate of MF, which progressively increased from 0.2 per 100,000, in the period 1995-1997, to 0.5 per 100,000 in the period 2010-2012 (17). The authors explained the increasing rates with the improvements in MF diagnosis and in data reporting following the latest WHO classification updates.

Several studies have shown that the incidence of MF increases with age (19, 21–23). The study by Hultcrantz et al. on data from the Swedish Cancer Register and the Swedish Blood Cancer Register has highlighted a substantially higher crude incidence rate of PMF in older versus younger individuals (19). Notably, the incidence was shown to increase from 0.04/100,000 person-years in the pediatric population to 0.67/100,000 person-years in the adult population aged 18-39 years and to 19/100,000 person-years in the elderly population aged > 70 years. Based on these findings, the authors pointed out that PMF may be more common than perceived among the elderly (19). A trend of progressive increase of PMF incidence with age has also been described in a recent review of data from the US SEER database showing an incidence per 100,000 person-years of 0.02 in individuals aged < 40 years and 2.0 in individuals aged ≥ 80 years (**Table 1**) (22).

### General characteristics of the myelofibrosis population

The first MPN Landmark survey in patients with MPN under treatment in the US addressed patient perception of disease burden and treatment management (31). Of the 813 respondents, 207 (25.5%) had MF; their median age was 66 years and the median duration of disease was 4 years. Almost half of MF patients had experienced at least one MF-related symptom ≥ 1 year before diagnosis. Fatigue and sleep problems were the most common symptoms reported by patients with MF. More than 80% of patients with MF reported a reduction of their quality of life due to MF-related symptoms (31).

The ERNEST registry (European Registry for Myeloproliferative Neoplasms; towards a better understanding of Epidemiology, Survival and Treatment) included the data of 1209 patients with MF (median age 66 years), 61% with PMF, 20% with post-ET MF, and 19% with post-PV MF (32). Data reported in 2014 revealed a certain variability in the rates of constitutional symptoms experienced (from 43% in post-ET MF to 49% in post-PV MF). Approximately 33.5% of patients died during follow-up (median duration 2 years); 8% of the cohort experienced leukemic transformation, with no substantial difference in the transformation rates in the three subgroups.

A further study of the ERNEST registry involving 1010 patients with MF (57.8% with PMF, 20.5% with post-ET MF, and 21.7% with post-PV MF; median age 63.7 years; 59.9% men) investigated the impact of ruxolitinib on overall survival using prospectively collected data. Age, male sex, high-risk category based on the Dynamic International Prognostic Scoring System (DIPSS) were identified as factors that negatively affected overall survival. Variables associated with favorable effects on overall survival included recent diagnosis and treatment with ruxolitinib (33).

In a multicenter observational study involving 408 patients with MF (54.4% had PMF, 27.7% post-ET MF, and 17.9% post-PV MF) treated with ruxolitinib in 18 hematology centers in Italy, median age was 68.5 years, and 56.4% were male (34). More than 80% of patients were classified as having intermediate-2/high risk according to the International Prognostic Scoring System (IPSS). Constitutional symptoms were present in 53.9% of patients. Median hemoglobin levels were 10.7 g/dL, 42.4% of patients had hemoglobin levels < 10 g/dL, and almost 30% of patients were transfusion-dependent. Platelet counts were > 200 x 10^9^/L in 63.5% of patients and < 100 x 10^9^/L in almost 10% of patients. Full molecular data were available for 79.2% of patients; the *JAK2* V617F mutation was the most common (87% of patients), followed by *CALR* mutations (8%) and *MPL* W515K/L (1%).

In a nationwide Japanese survey about features and outcomes of 780 patients with PMF (median age 66 years, 67% male), altered blood cell counts and other laboratory abnormalities were the main reason for being referred to a hematologist, while only about 20% of patients presented constitutional symptoms (35). Splenomegaly was found in 75% of patients. Fifty-six percent of patients who were tested for *JAK2* mutational status had the V617F mutation.

The analysis of the MERGE registry including patients with MPNs from Asia, Middle East, Turkey, and Algeria showed that 67.5% of the 169 patients with MF harbored the *JAK2* V617F mutation; 49.7% were male and their median age was 58.8 years (36). Constitutional symptoms were significantly more common and severe in patients with MF.

According to a recent analysis of the Spanish Registry of Myelofibrosis, including 1000 patients diagnosed with PMF (641) and secondary MF (359), the most common symptoms at presentation were moderate to severe anemia (Hb < 10 g/dL) (36%), constitutional symptoms (35%), and symptomatic splenomegaly (17%) (37). The cumulative incidence of leukemic transformation at 10 years was 15%. In a subsequent analysis of the same registry (1613 patients with MF), palpable hepatomegaly was present in 19.3% of patients at diagnosis (8). The hemoglobin level was < 10 g/dL in 33.1% of patients; platelet counts indicative of severe thrombocytopenia (< 50 x 10^9^/L) were found in 4.8% of patients, while 8.7% of patients had platelets counts of 50-100 x 10^9^/L. Mutations were detected in the following genes: *JAK2* (71.4%), *CALR* (17.7%), and *MPL* (4.2%) (8).

### Myelodepletive phenotype of myelofibrosis

Disturbances in hematopoiesis associated with MPNs can lead to two phenotypes, the myeloproliferative phenotype and the myelodepletive phenotype, which are the two extreme manifestations of the entire disease spectrum (38). The myeloproliferative phenotype is characterized by peripheral elevated blood cell counts and is associated with constitutional symptoms and progressive splenomegaly (38). The myelodepletive phenotype is characterized by cytopenias and lower JAK2 variant allele frequency (39) and patients with this phenotype often require transfusions and have an increased risk of infections and bleeding, with a negative impact on survival (38). The distinction of the two phenotypes is important also for therapeutic decisions. In fact, patients with the myelodepletive phenotype may not benefit from the treatment with ruxolitinib and may therefore need alternative strategies (38). The lack of benefits from ruxolitinib in MF patients with the myelodepletive phenotype was recently highlighted by a retrospective analysis of 886 patients with MF, 45.9% of whom were cytopenic (40). As a consequence, inferior outcomes in the myelodepletive phenotype could be due, at least partially, to lack of valid therapies (except for transplant) and/or suboptimal dosage of available drugs due to cytopenias.The myelodepletive phenotype is more prevalent in PMF than in other MPNs (38). A recent study investigating this phenotype in prefibrotic versus overt PMF found that cytopenias were more common in overt PMF and had a distinct prognostic value in the two disease subtypes (41). While the myelodepletive phenotype was associated with shorter overall survival in both subtypes, in patients with prefibrotic PMF it was associated with an increased risk of leukemic transformation (41). In addition, in prefibrotic PMF the myelodepletive phenotype was found to be a risk factor also for fibrotic progression.

Within the myelodepletive phenotype, severe thrombocytopenia, defined by < 50 x 10^9^ platelets/L, has the worst impact on prognosis (42). Recent studies in patients with MF have shown that the rates of anemia and transfusion need are highest in those with severe thrombocytopenia; these patients also have a greater risk of leukemic transformation and shorter overall and leukemia-free survival (8, 42). While the incidence of severe thrombocytopenia in patients with MF at diagnosis has been estimated to range from 11% to 16% (43), a series of international surveys among physicians (n = 807) have consistently revealed that the prevalence of this conditions in the MF population is higher and approximately 35% (43).

### Thrombosis and bleeding

Patients with MPNs, including PMF, have an increased risk of thrombotic and thromboembolic events compared to the general population (44–47). At the same time, due to disease-related factors as well as to antiplatelet and anticoagulant therapy used to prevent thrombosis, MPN patients are at increased risk of bleeding (46, 48). Thrombosis and bleeding contribute significantly to the morbidity and mortality of MPNs (46, 47). These events can occur before or at the time of MPN diagnosis, and arterial thrombosis appears to be more common than venous thrombosis (49, 50). Of note, a recent population-based retrospective study found that MF is associated with an increased risk of venous thromboembolism but not of arterial thromboembolism (49). In about 20% of the cases of MPN, thrombosis is the first event that leads to the diagnosis of MPN (51). The risk of thrombosis can persist during the follow-up, with the highest incidence in patients with PV (3.5/100 person-years), while the incidence in patients with ET or PMF is lower (2.5/100 person-years) (51). An analysis of data from the MPN registry of the German Study Alliance Leukemia (SAL) showed that approximately one-third of patients with MPN experience a vascular event (46). According to a recent meta-analysis of 29 studies (13,436 patients with MPNs), the pooled prevalence of overall thrombosis among patients at diagnosis was 20.0%; the pooled prevalence of arterial thrombosis was 16.2% and the pooled prevalence of venous thrombosis was 6.2% (48). Common thrombotic events were cerebrovascular disease, coronary heart disease, and deep venous thrombosis. The pooled prevalence of bleeding events (mostly gastrointestinal, mucosal, and cutaneous bleeding) among patients newly diagnosed with MPNs was 6.2%. patients newly diagnosed with PMF, the pooled overall prevalence of thrombosis was 9.5%; the pooled prevalence of bleeding at diagnosis was 8.9% (48). A recent study on the data from the Spanish Registry of Myelofibrosis found cumulative rates of thrombosis and bleeding of 6.4% and 5.3%, respectively, resulting in incidence rates of 1.65 events/100 patient-years and 1.5 events/100 patient-years, respectively (8). **Table 2** summarizes current knowledge of the epidemiology of thrombosis and bleeding in patients with MF.

**Table 2:** Epidemiology of thrombosis and bleeding in myelofibrosis.

Study	Country and period	Population	Thrombosis	Bleeding
Barbui et al., 2010 (44)	Italy and Spain, 1973-2008	▪ Pts databases of the four institutions participating in the study ▪ n=707 with PMF	▪ 7.2% of pts had fatal and nonfatal thromboses ▪ Estimated rate: 1.75% pts/yrs	na
Kaifie et al., 2016 (46)	Germany, 2012-2015	▪ MPN Registry of the German Study Alliance Leukemia ▪ n=454 with MPN; 113 (24.9%) with PMF; 22 (4.8%) with post-PV MF; 19 (4.2%) with post-ET MF	▪ 31.2% of pts with PMF had a vascular event. The most frequent events were deep vein thrombosis (31.3%), stroke (21.2%) acute coronary syndrome (20.6%), and splanchnic vein thrombosis (18.2%). ▪ 61.9% and 42.1% of pts with post-PV MF and post-ET MF, respectively, had a vascular event. The most frequent events were deep vein thrombosis and acute coronary syndrome	▪ 9.3% of pts with PMF had a bleeding event ▪ 19.1% and 5.3% of pts with post-PV MF and post-ET MF, respectively, had a bleeding event
Hultcrantz et al., 2018 (45)	Sweden, 1987-2009 with follow-up to 2010	▪ Swedish Cancer Register ▪ n=9429 with MPN and 35,820 matched controls; 1488 (15.8%) with PMF	▪ Significantly increased rate of thrombosis in MPN pts vs controls at diagnosis and shortly after ▪ HR for thrombosis of MPN pts at 3 months after diagnosis: 4.0 (95% CI 3.6-4.4); for MPF pts: 4.7 (95% CI 3.6-6.0)	na
Lindgren et al., 2022 (47)	Sweden	▪ Swedish MPN Registry ▪ n=392 pts with MF	▪ 58 pts (14.8%) with vascular complications, mostly thrombotic ▪ Incidence of vascular events: 2.8/100 patient-yrs ▪ Survival of pts with vascular complication significantly shorter vs matched controls	na
Hernández-Boluda et al., 2022 (52)	Spain, 2000-2021	▪ Spanish Myelofibrosis Registry ▪ n=1613 pts with MF; 15.4% had previous PV; 24.2% had previous ET ▪ Median follow-up after diagnosis: 6.7 yrs	▪ 14% with history of thrombosis at diagnosis ▪ 6.4% had ≥ 1 thrombotic event during follow-up ▪ Incidence rate: 1.65/100 patient-yrs for both PMF and secondary MF. The overall incidence rate of arterial and venous thrombosis was 0.97 and 0.67 events per 100 patient-years, respectively.	▪ 3.1% with bleeding at diagnosis ▪ 5.3% had ≥ 1 major bleeding event during follow-up ▪ Incidence rate: 1.5/100 patient-yrs
Saliba et al., 2020 (49)	Israel, 2007-2017	▪ Database of largest healthcare provider ▪ n=642 pts with MF; 2568 matched controls	▪ Incidence rate of venous thromboembolism: 11.4/1000 patient-yrs (2.2 in controls) ▪ Incidence rate of arterial thromboembolism: 24.7/1000 patient-yrs (15.9 in controls) ▪ Adjusted HR for venous thromboembolism: 6.88 (95% CI 2.02-23.45) ▪ Adjusted HR for arterial thromboembolism: 0.94 (95% CI 0.49-1.77)	na
Song et al., 2021 (50)	Korea, 1996-2020	▪ Single-centre retrospective cohort ▪ n=335 pts with MPN; 72 with pre-PMF/PMF (21.5%)	▪ Rate of thrombotic events: 38.1% in pts with pre-PMF and 13.3% in pts with PMF ▪ Arterial thrombosis more frequent than venous thrombosis ▪ Most events were reported before/at diagnosis	▪ Rate of bleeding events: 9.5% in pts with pre-PMF and 3.3% in pts with PMF

ET, essential thrombocythemia; HR, hazard ratio; MF, myelofibrosis; MPN, myeloproliferative neoplasm; na, not available; PMF, primary myelofibrosis; pts, patients; PV, polycythemia vera; vs, versus; yrs, years.

### Risk of infections

Infections are common consequences of cytopenias and are among the leading causes of death of patients with MF (2). A recent population-based study assessed the risk of serious infections in > 8000 patients with MPNs versus > 32,000 matched controls (53). The study estimated hazard ratios of 2.0 for any infections, 1.9 for bacterial infections, and 2.1 for viral infections. Patients with PMF had the highest increase in the risk of any infections (HR, 3.7). There were no statistically significant differences in the risk of infections between untreated and treated patients (with interferon-α or hydroxyurea), suggesting that the increased risk of MPN patients was disease-related (53). A retrospective analysis of the data of 446 MF patients treated with ruxolitinib (median exposure to ruxolitinib, 23.5 months) in 23 European centers found that 28% of patients experienced infectious events, mostly involving the airways, with an estimated incidence rate of 17 events per 100 patient-years (54). A recent pilot, patient-reported study involving centers from Germany and Italy, administered a questionnaire to 948 patients with MPNs (23.5% with MF) enquiring about infectious events over the past 12 months (55). Overall, 50.5% of all patients reported ≥ 1 infectious events; in the MF subgroup, 57.4% reported ≥ 1 infectious events (p = 0.022). The risk of infections was increased also in patients treated with ruxolitinib (68.2% reported ≥ 1 infectious events (p = 0.01). This pilot study also highlighted that preventive measures to control infections were suboptimal (55).

### Prefibrotic and overt primary myelofibrosis

Prefibrotic MF is clearly distinct from overtly fibrotic PMF (3, 56, 57). It is now generally accepted that prefibrotic MF is also distinct from ET (3, 56, 57). Evidence has clearly shown that prefibrotic PMF has a worse prognosis than ET, with an increased risk of bleeding and a greater propensity to progress to overt fibrosis and leukemic transformation (56–59).

An Italian real-life study in 661 patients with PMF (42% with prefibrotic PMF and 58% with overtly fibrotic PMF) investigated the clinical and molecular features of prefibrotic PMF and overtly fibrotic PMF (60). Driver mutations were equally distributed between the two PMF forms; however, overt PMF was more frequently associated with an unfavorable karyotype, as well as a more severe symptom burden. Patients with overtly fibrotic PMF had a shorter survival compared with patients with prefibrotic PMF (7.2 vs 17.6 years) (60). Given the similarly increased rate of thrombotic events reported in prefibrotic PMF patients and ET patients, a recent study investigated whether the international prognostic score for thrombocytosis in essential thrombocytopenia (IPSET) score, developed for the evaluation of thrombotic risk in patients with ET, could be used also in patients with prefibrotic PMF (61). The study involved 328 patients with prefibrotic PMF, with incidence rates of arterial and venous thromboses after diagnosis of 1.0 and 0.95/100 patient-years, respectively. Age, leukocytosis, cardiovascular risk factors, *JAK2* V617F mutation and other high-risk mutations were identified as significant predictors of arterial thrombosis in these patients; the only predictor of venous thrombosis was a history of thrombosis, particularly venous thrombosis. The study demonstrated that the IPSET risk-stratification system was accurate also for risk assessment in prefibrotic PMF (61).

Treatment outcomes of patients with prefibrotic and overt PMF seem to differ. An Italian study in 232 patients treated with ruxolitinib compared treatment outcomes in patients with prefibrotic PMF and overt PMF and found that patients with prefibrotic disease had better and more sustained responses than patients with overt disease; the profile of hematologic toxicities was more favorable in patients with prefibrotic PMF (62). There were however no differences between the two groups in overall and leukemia-free survival (62). The impact of fibrosis grade on response and outcomes of PMF patients treated with ruxolitinib was further investigated in a *post-hoc* analysis of the JUMP study (63). At baseline, patients with higher-grade fibrosis were more affected by anemia and thrombocytopenia than patients with lower-grade fibrosis; PMF symptoms and splenomegaly were comparable in the two groups. Spleen responses to treatment and survival tended to be better for patients with lower-grade fibrosis, suggesting that early start of ruxolitinib may be associated with greater benefits.

### Secondary myelofibrosis

About 15% of patients with ET or PV develop a PMF-like phenotype, referred to as post-ET or post-PV MF, or secondary MF (2). A review about the rates of fibrotic progression in PV and ET found a cumulative incidence of secondary MF at 10 years of 4.9-6% for PV and 0.8-4.9% for ET (64). Overall, secondary MF is treated like PMF with apparently similar outcomes (2). However, evidence suggests that post-ET and post-PV MF differ from PMF in terms of prognosis (65). In fact, the DIPSS, developed and validated for PMF, was shown to be unable to identify higher-risk patients among individuals with post-PV and post-ET MF (65). A validated, specific tool for evaluating the risk of progression and predicting survival in secondary MF – the MYSEC prognostic model – is available (66, 67).

A study in 421 patients with post-ET or post-PV MF treated with ruxolitinib evaluated the disease phenotype and the response and toxicity to ruxolitinib (66). Compared to PMF, post-PV and post-ET MF were characterized by increased cell proliferation at diagnosis and at the beginning of ruxolitinib treatment. The response rates to ruxolitinib were similar between PMF and secondary MF; however, the rates of ruxolitinib-related anemia and thrombocytopenia were lower in post-PV and post-ET MF than in PMF (66). A study involving the patient cohort of the MYSEC project (n = 1258) assessed the incidence of thrombosis in secondary MF and investigated predictors of thrombotic events (68). Over a median follow-up of 3.5 years, 10.7% of patients had a thrombotic event, corresponding to an incidence rate of 2.3/100 patient-year. Cytoreductive treatment, both with hydroxyurea and with ruxolitinib, was associated with fewer thrombotic events compared with no treatment.

A study in 589 patients with MF, including both primary and secondary MF, evaluated the impact of ruxolitinib therapy on the evolution to the blast phase (69). After a median follow-up of 3 years from ruxolitinib start, 11% of patients had progressed to the blast phase; most of them (93.8%) had progressed during treatment. The incidence rate of progression to the blast phase was 3.7 per 100 patient-years, and similar in PMF and secondary MF. The risk scoring systems DIPSS and MYSEC accurately predicted the progression to the blast phase in patients with PMF and secondary MF, respectively (69).

Finally, in a study on 359 patients with post-PV and post-ET MF, the Italian cooperative group AGIMM (AIRC-Gruppo Italiano Malattie Mieloproliferative) described the mutation profile of secondary MF and its clinical impact (70). The allele burden of *JAK2* V617F and *CALR* mutations was found to be significantly greater in post-PV and post-ET MF compared with PV and ET, with however no effects on overall survival. As for other mutations belonging to the category of high molecular risk (HMR) in PMF, namely *ASXL1*, *EZH2*, *SRSF2*, *IDH1*, and *IDH2*, only *SRSF2* mutations were associated with reduced survival in post-ET MF. These findings suggested that other mutations may be implicated in secondary MF (70).

### Patterns of myelofibrosis management

Treatment decisions in patients with MF are made based on the estimated risk of disease progression and death. As mentioned in the previous sections, various validated prognostic systems are available for the assessment of risk, including the IPSS (International Prognostic Scoring System) (71), DIPSS (Dynamic International Prognostic Scoring System) (72), DIPPS-plus (73) and the newer prognostic models MIPSS70 (Mutation-enhanced IPSS for patients aged ≤ 70 years) (74), MIPSS70+ version 2.0 (the karyotype-enhanced MIPSS70), and GIPSS (the genetically-inspired IPSS, which depends exclusively on mutations and karyotype) (75) (**Table 3**). Some of these scores (IPSS, DIPSS, DIPSS-plus) have proven useful also for selecting MF patients who may benefit from allogeneic hematopoietic cell transplantation (allo-HCT), so far the only curative approach. A clinical-molecular MF transplant scoring system (MTSS) predicting posttransplant outcomes in patients with PMF or secondary MF undergoing allo-HCT has been recently developed and validated (**Table 3**) (76).

**Table 3:** Tools for risk assessment in patients with primary myelofibrosis.

Prognostic score	Features considered	Risk categories (Median Survival)
**International Prognostic Scoring System** **IPSS** (71)	▪ Age ≥ 65 yrs ▪ Hemoglobin < 10 g/dL ▪ Leucocyte count > 25 x 10^9^/L ▪ Circulating blasts ≥ 1% ▪ Constitutional symptoms To be used at diagnosis	▪ Low: no features (11.3yrs) ▪ Intermediate-1: 1 feature (7.9yrs) ▪ Intermediate-2: 2 features (4yrs) ▪ High: ≥ 3 features (2.3yrs)
**Dynamic International Prognostic Scoring System** **DIPSS** (72)	▪ Age ≥ 65 yrs ▪ Hemoglobin < 10 g/dL ▪ Leucocyte count > 25 x 10^9^/L ▪ Circulating blasts ≥ 1% ▪ Constitutional symptoms To be used at diagnosis and during clinical course of disease; a score of 2 is assigned to hemoglobin < 10 g/dL; a score of 1 is assigned to all other features	▪ Low: score 0 (NR) ▪ Intermediate-1: score 1-2 (14.2yrs) ▪ Intermediate-2: score 3-4 (4yrs) ▪ High: score 5-6 (1.5yrs)
**Dynamic International Prognostic Scoring System-Plus** **DIPSS-Plus** (73)	▪ Age ≥ 65 yrs ▪ Hemoglobin < 10 g/dL ▪ Leucocyte count > 25 x 10^9^/L ▪ Circulating blasts ≥ 1% ▪ Constitutional symptoms ▪ Unfavorable karyotype ▪ Need for red cell transfusion ▪ Platelet count < 100 x 10^9^/L	▪ Low: no features (15yrs) ▪ Intermediate-1: 1 feature (6.5yrs) ▪ Intermediate-2: 2-3 features (2.9yrs) ▪ High: ≥ 4 features (1.3yrs)
**Mutation-enhanced International Prognostic Scoring System for patients aged ≤ 70 yrs (transplant-age patients)** **MIPSS70** ^a^ (74)	▪ Absence of *CALR* type 1/like mutation ▪ Presence of high molecular risk mutations ▪ Presence of ≥ 2 high molecular risk mutations ▪ Hemoglobin < 10 g/dL ▪ Leucocyte count > 25 x 10^9^/L ▪ Platelet count < 100 x 10^9^/L ▪ Circulating blasts ≥ 2% ▪ Bone marrow fibrosis grade ≥ 2 ▪ Constitutional symptoms A score of 2 is assigned to leucocyte count > 25 x 10^9^/L, platelet count < 100 x 10^9^/L, and presence of ≥ 2 high molecular risk mutations; a score of 1 is assigned to all other features	▪ Low: score 0-1 (27.7yrs) ▪ Intermediate: score 2-4 (7.1yrs) ▪ High: score ≥ 5 (2.3yrs)
**Genetically-inspired International Prognostic Scoring System** **GIPSS** (75)	▪ VHR (very high risk) karyotype ▪ Unfavorable karyotype ▪ Absence of *CALR* type 1/like mutation ▪ Presence of *ASXL1*, *SRSF2*, and *U2AF1Q157* mutations A score of 2 is assigned to VHR karyotype; a score of 1 is assigned to all other features	▪ Low: score 0 (26.4yrs) ▪ Intermediate-1: score 1 (8yrs) ▪ Intermediate-2: score 2 (4.2yrs) ▪ High: score ≥ 3 (2yrs)
**Myelofibrosis transplant scoring system** **MTSS** (76)	▪ Age ≥ 57 yrs ▪ Karnovsky score < 90% ▪ Platelet count < 150 x 10^9^/L ▪ Leucocyte count > 25 x 10^9^/L ▪ HLA mismatched donor ▪ *ASXL1* mutation ▪ non-*CARL*/*MPL* genotype A score of 2 is assigned to HLA mismatch and non-*CARL*/*MPL* genotype; a score of 1 is assigned to all the other features	▪ Low: score 0-2 (5yrs OS: 90%) ▪ Intermediate: score 3-4 (5yrs OS: 77%) ▪ High: score 5 (5yrs OS: 50%) ▪ Very high: > 5 (5yrs OS: 34%)

a Updated versions of this scoring system, MIPSS70+ and MIPSS70+ version 2.0, are available (75).

The discussion of currently available and recommended treatments is beyond the scope of this article. For information on recommended therapies and allo-HCT, the reader is referred to the latest international guidelines (2, 5, 77).

### Real-world management

The GIMEMA (Gruppo Italiano Malattie EMatologiche dell’Adulto) Myeloproliferative Neoplasms Working Party performed a survey among 950 hematologists treating MPN patients about diagnostic procedures, risk stratification, management, and therapeutic choices in Italy (78). Overall, 180 hematologists (18.9%) completed the survey. The results of the survey showed that driver mutations were tested by 88.3% of hematologists when there is a suspicion of PMF; karyotype analysis of MF patients is performed by 71.1% of hematologists. Most physicians (97.9%) defined high-risk MF based on age > 60 and/or previous history of major vascular event. Validated prognostic scoring systems were used at diagnosis by 63.9% (IPSS) and 7.6% (DIPSS/DIPSS-plus) of hematologists. Routine testing of HMR-mutations was performed at diagnosis by less than one-third of physicians. Also, less than 5% of physicians used the MIPSS70 score at diagnosis. Ruxolitinib was the first-line treatment of splenomegaly for 57.2% of respondents followed by hydroxyurea (42.8%). The survey highlighted a notable homogeneity of MF management across the country with no substantial differences in practice between hospital- and academia-based Italian hematology centers. The compliance to international recommendations was also remarkable.

Another Italian survey, led by the Italian MPN Lab collaboration, investigated the diagnostic evaluation, prognostic assessment, and use of ruxolitinib in real-life clinical practice in 18 hematology centers in Italy (79). This survey found that risk scores requiring the evaluation of non-driver HMR mutations (ie, MIPSS70, MIPSS70+ v2, and GIPSS) were not routinely used. The reasons for the limited use of the newest, genetically-inspired prognostic systems were the lack of sequencing facilities and concerns about the costs of these molecular analyses. Spleen size was commonly assessed by palpation, with no radiological confirmation. Ruxolitinib treatment appeared to be administered according to current recommendations: screening for previous hepatitis B and C infections and latent tuberculosis was commonly performed prior to treatment start. However, there was poor agreement about the criteria that define spleen response to ruxolitinib. A shared definition of treatment failure was also lacking, according to the participants in the survey. A second survey led by the Italian MPN Lab collaboration addressed the unresolved question concerning the response to MF treatment and the treatment with JAK inhibitors of specific patients groups typically encountered in clinical practice, including patients with anemia, thrombocytopenia, and infections (80). The results of the survey highlighted the need for a shared definition of response to treatment and guidelines for the management of patients with concomitant conditions that may complicate treatment (80). The interim analysis of the ROMEI study, a real-life, prospective, observational study in MF patients treated with ruxolitinib in Italy, showed that about one-third of patients may be undertreated due to poor adherence to oral therapy (81). In this observational study, treatment adherence was assessed using the 8-item Morisky Medication Adherence Scale (MMAS-8). The preliminary findings from another Italian study (RAMP) assessing the adherence to ruxolitinib in MF and PV patients with the Adherence to Refills and Medications Scale (ARMS) revealed low adherence in 51.8% of MF patients and a negative impact of low adherence on spleen response (82).

Interesting data about the real-world management of MF are available also from other countries. A retrospective chart review (491 patients diagnosed with MF between 2012 and 2016) was performed in the US to investigate how physicians assess the risk of patients at diagnosis (83). The review found that risk categorization was not reported in 30.1% of patients. A formal risk scoring system was used in only 49.9% of patients whose risk was evaluated. More than 40% of physician-assigned risk categorizations were incorrect, compared to risk assessment with validated prognostic systems, leading in most cases to an underestimate of the risk. Furthermore, a relevant proportion of patients (38.5%) did not initiate treatment promptly after diagnosis, despite having been formally categorized as intermediate- or high-risk (83).

A study using data from the US SEER database evaluated the patterns of care of older patients with MF before and after ruxolitinib approval (528 patients with MF and a median age at diagnosis of 76 years over the period 2007-2015) (84). Of the 298 patients who were diagnosed after ruxolitinib approval (2012-2015), 113 (37.9%) received ruxolitinib. Approximately half of the patients prescribed ruxolitinib also took hydroxyurea and/or prednisone. The median duration of ruxolitinib treatment was 11.9 months. The results of this US survey suggest that treatment with ruxolitinib still needs to be optimized (84).

The retrospective study REALISM investigated early management of patients with MF in the UK (85). The primary endpoint of the study was the time from diagnosis to active treatment. The study included 200 patients, 63% with primary MF and 37% with secondary MF. The study revealed insufficient documentation of symptoms and prognostic scores at diagnosis; patient reported outcomes were rarely used. Notably, the watch-and-wait strategy was used in more than half of the patients (53.5%), including patients with intermediate-2/high IPSS risk scores. However, a progressively decreasing trend in the use of the watch-and-wait strategy was observed from 2013 to 2017. The most frequently prescribed treatments were hydroxyurea and ruxolitinib. The median time to first active treatment was 46 days. Overall, this UK study highlights that several aspects of real-life management of patients with MF need to be improved.

### Myelofibrosis and COVID-19

The pandemic of coronavirus disease 2019 (COVID-19) has had a tremendous impact on healthcare systems and the management of patients with chronic conditions. Cancer patients, and especially those affected by hematologic malignancies, have emerged as a vulnerable group with an increased risk of developing severe/critical COVID-19 compared with the general population (86, 87). In May 2020, an observational study involving 38 centers across Italy, Spain, Germany, Poland, France, and the UK was launched by the European Leukemia Net (ELN); the study included 175 patients with MPNs and COVID-19 (88). According to this study, the mortality of MF patients with COVID-19 was 48%, after a median follow-up of 50 days. Significant predictors of death among the study population included male gender, older age, decreased lymphocyte counts, need for respiratory support, comorbidities, and diagnosis of MF. Although treatment with ruxolitinib did not seem to have an effect on mortality, its abrupt discontinuation was associated with an increased risk of death (88). This effect is explained by the fact that the sudden withdrawal of ruxolitinib may lead to very rapid increase of inflammatory cytokines; this, in turn, can have lethal consequences in the presence of the hyperinflammatory status associated with severe COVID-19 (88). The management of patients with MF in the setting of a COVID-19 pandemic is complex (88, 89). Ruxolitinib may increase the risk of infections, but its withdrawal should be carefully considered in patients with MF and concomitant COVID-19. MF patients are at increased risk of thrombotic and bleeding events, which are also common complications of severe/critical COVID-19 (88, 89). All MPN patients should receive COVID-19 vaccination (89). However the use of ruxolitinib may be associated with impaired immune response. A study in 42 patients with MPNs on active treatment found significantly lower rates of seroconversion and seroprotection in patients with MF, compared with patients with PV or ET, after the second dose of the mRNA vaccine BNT162b2 (90). The use of ruxolitinib might have contributed to the lower immune response of MF patients, according to the authors. These findings point out that patients with MF, especially those on treatment with ruxolitinib, need personalized vaccination schedules and additional protective measures against SARS-CoV-2 infection (88, 89, 91). The combination of the anti-SARS-CoV-2 monoclonal antibodies tixagevimab and cilgavimab, approved for preexposure prophylaxis of SARS-CoV-2 infection, may be used in patients with MF for passive immunization (92).

The COVID-19 pandemic and the consequent strain on healthcare systems have led to the rapid development and implementation of novel strategies for patient management. Telemedicine, for example, has been used in many areas of medicine that require regular patient follow-up, with positive results, as reported by a survey among 365 Italian patients with MPNs whose visits were performed via telephone calls between March and May 2020 (93).

### Tools and strategies for improving the management of myelofibrosis

Well-designed, population-based registries have proven to be a very important source of high-quality data (19, 27, 46, 94). They not only provide epidemiologic information but can also be used for comparing treatments and outcomes in the real-life setting and improving the standards of care. There is a strong need for high-quality, standardized registries especially for rare conditions including MF. Efforts to design, implement, and maintain such registries should be strongly encouraged.

Large surveys involving clinicians and patients are another tool that has proven useful for getting insights into real-life issues related to the management of MF. This strategy was successfully used in the US MPN Landmark Survey of MPNs (31). This approach has allowed to focus on patient-reported outcomes, as well as on clinicians needs and attitudes, that are often not included among the endpoints of clinical trials. In Italy, a number of relevant surveys in the field of MPNs have been conducted by GIMEMA (Gruppo Italiano Malattie Ematologiche dell’Adulto) MPN Working Party, including the survey about the management of MPNs in clinical practice in Italy discussed in the previous section (78). During the COVID-19 pandemic, GIMEMA performed surveys among clinicians to explore how the pandemic was changing the attitude of hematologists towards MPNs and to determine the prevalence of SARS-CoV-2 infection in MPNs patients in Italy, as well as the outcomes of infected patients (79, 95, 96).

Social media, usually Twitter, have become a widespread tool for communications also among scientists and clinicians (97). In the field of MPNs, the creation in 2015 of the disease-specific hashtag #MPNSM directed to healthcare providers confirmed the feasibility of this approach and was associated with increased communication among users (97). Interestingly, a survey by the creators of #MPNSM among patients with MPNs about their use of online resources found that patients had different social media preferences compared with physicians: while physicians rapidly adopted Twitter for medical communication, MPN patients showed a preference for Facebook, Google/Google+, YouTube, and blogs (98).

## Discussion and conclusions

With this review we aimed to provide an overview of current knowledge about the epidemiology of MF and to discuss relevant aspects related to the recognition and management of this condition in clinical practice, with an emphasis on Italy. Several issues have emerged from the present review. First of all, epidemiologic data about MPNs continue to be limited. There are only a few studies devoted entirely to MF. To the best of our knowledge, there is no updated epidemiologic information about MF patients in Italy. Only a few countries appear to have long-standing and well-established registers of MPNs data. Overall, the need for standardization of data collection and reporting continues to be relevant and insufficiently addressed.

MF is clearly a disease of older age. Patients with MF are typically aged > 65 years, often present with unexplained abnormalities in blood counts, and have a substantial burden of symptoms, especially constitutional symptoms. They are at increased risk of infections, thrombosis and bleeding. Thrombosis and bleeding can be present before/at diagnosis in up to one-fifth of cases.

In recent years, there has been an increased awareness of distinct MF forms, with practical implications. The distinction of the two phenotypes of MF, the myeloproliferative and myelodepletive phenotypes, is important for treatment decisions because patients with the myelodepletive phenotype may not benefit from therapies that exacerbate cytopenias. Furthermore, PMF can be separated based on fibrosis grade into prefibrotic disease and overtly fibrotic disease; prefibrotic disease responds better to current treatments, while overtly fibrotic disease has a worse prognosis. Our understanding of the differences between PMF and secondary MF is also improving. These differences need to be stressed because PMF and secondary MF appear to have a different prognosis and may benefit from distinct treatment approaches. A validated prognostic tool is available for risk assessment of patients with secondary MF – the MYSEC scoring system – the use of which should be encouraged.

The picture provided by surveys on the real-world management of MF in Italy is encouraging and shows and overall good compliance to international recommendations and the use of validated prognostic scoring systems at diagnosis (IPSS, DIPSS, DIPSS-plus). There is however a lack of shared criteria that define response to treatment and treatment failure. Also lacking are guidelines for the management of MF patients with conditions, like infections and cytopenias, that may complicate treatment.

The COVID-19 pandemic had posed additional challenges to the management of MF. Patients with MF, who are recognized as a group at high risk of developing severe-critical COVID-19, need personalized vaccination schedules and additional protective measures.

To address all the unmet issues highlighted by the present review, the authors strongly suggest the following measures:

- Diagnostic procedures need to be improved and standardized both for PMF and secondary MF. Bone marrow biopsy and bone marrow aspiration should be performed by a clinician with expertise in MF. Besides bone marrow examination, diagnosis should include karyotype and driver mutation analysis.

- Regular follow-up is crucial for the early detection of signs of disease progression.

- MF management needs to be more consistent across centers.

- Early access to the evaluation for transplantation eligibility should be available to all patients. In addition, given the advances in transplant procedures and the availability of therapies that are significantly improving the clinical status of patients with MF, efforts are needed to extend the use of allo-HCT to more patients.

- Shared criteria that define response to treatment and treatment failure are urgently needed.

- National networks have proven valuable in many therapeutic areas and their creation should be encouraged and supported also in the setting of MF. Such networks could contribute to the collection of epidemiologic data and help define the prevalence of MF.

- National network could also help create shared and standardized treatment protocols and may facilitate the access of patients to novel treatment options.

## Author contributions

MBr: Writing – original draft, Writing – review & editing, Conceptualization, Supervision. FP: Writing – original draft, Writing – review & editing, Conceptualization, Supervision. NP: Writing – original draft, Writing – review & editing, Conceptualization. MC: Writing – original draft, Writing – review & editing, Supervision. MBe: Writing – original draft, Writing – review & editing, Supervision. GP: Writing – original draft, Writing – review & editing, Conceptualization, Supervision. VS: Writing – original draft, Writing – review & editing, Conceptualization, Supervision.
